# Comparison of a Sleep Item From the General Health Questionnaire-12 With the Jenkins Sleep Questionnaire as Measures of Sleep Disturbance

**DOI:** 10.2188/jea.JE20110023

**Published:** 2011-11-05

**Authors:** Tea Lallukka, Alexandru Dregan, David Armstrong

**Affiliations:** 1 Hjelt Institute, Department of Public Health, University of Helsinki, Helsinki, Finland; 2 Department of Sociology, University of Surrey, Guildford, UK; 3 Department of Primary Care and Public Health Sciences, King’s College London, London, UK

**Keywords:** sleep, validation, GHQ-12, JSQ, single-item, multi-item

## Abstract

**Background:**

The objective of this study was to examine whether a widely available single-item measure of sleep disturbances is an acceptable alternative to a multi-item sleep questionnaire.

**Methods:**

Data were derived from Finnish Helsinki Health Study postal questionnaires administered in 2000–2002 (*n* = 7777, response rate 67%). The measures were the 4-item Jenkins Sleep Questionnaire (JSQ) on difficulties initiating and maintaining sleep, and nonrestorative sleep, and an item on sleep loss due to worry, from the General Health Questionnaire-12 (GHQ-12). Receiver operating characteristics (ROC) curve analyses were done to compare the predictive performance of the GHQ-12 item with the JSQ scale. Using the above 2 measures of sleep, logistic regression models were used to examine associations between sociodemographic factors, working conditions, health-related factors, and sleep disturbance.

**Results:**

The estimated area under the ROC curve was 0.68 among both women and men, which suggests that the ability of the GHQ-12 item to discriminate true positives from false positives was modest. However, the associations of sleep disturbance with its key determinants were largely similar using the GHQ-12 and the JSQ.

**Conclusions:**

A widely available, GHQ-12-based, single-item sleep measure was not an adequate substitute for a multi-item measure of overall sleep disturbance. Although the measures produced largely similar associations for key determinants of poor sleep, the discrepancies between responses must be considered when analyzing data from a measure that uses a single sleep item.

## INTRODUCTION

Due to the importance of sleep and sleep loss to health and well-being, a number of questionnaires to measure sleep disturbance have been developed. The Pittsburgh Sleep Quality Index (PSQI)^1^ and the Jenkins Sleep Questionnaire (JSQ)^2^ are 2 of the most commonly used multiple-item scales. The JSQ was developed to examine sleep problems in clinical research and has been validated among air traffic controllers and patients recovering from cardiac surgery.^2^ It is frequently used in epidemiologic studies^3^^,^^4^ and has good internal reliability.^2^ Sleep disturbance is also sometimes assessed using 1 item, such as the single question from the well validated General Health Questionnaire-12 (GHQ-12).^5^^,^^6^

In general, multiple-item measures are better at capturing complex underlying constructs, but single items are easier to collect and are cost-effective in large surveys.^7^ However, if a single item, such as the one derived from the GHQ-12, were a suitable alternative for assessing sleep disturbances, there would be more opportunities to focus on the causes and consequences of sleep disturbance in representative epidemiologic studies. In addition, studies that used different measures could be compared. Evidence on characteristics that explain different dimensions of sleep disturbance is equally important when interpreting results from studies that have used such single-item or multi-item measures of sleep disturbances.

This study examined the suitability of using a single-item measure, derived from the GHQ-12, to assess overall sleep disturbance, as determined by the JSQ, a validated and widely used measure of sleep disturbance.^2^ The more-specific aims were to examine whether associations between key predictors of sleep disturbance are similar when comparing single-item and multi-item outcome measures and to characterize respondents with discrepant responses to the 2 sleep measures.

## METHODS

### Data

The study analyzed data from the Helsinki Health Study 2000–2002 surveys of employees of the City of Helsinki, Finland aged 40 to 60 years (*n* = 8960).^8^ The response rate to the survey was 67%, and the data were broadly representative of the target population.^9^^,^^10^

### Sleep measures

The Helsinki Health Study used the JSQ to measure sleep disturbance over the preceding 4 weeks.^2^ This questionnaire consists of 4 items rated on a 6-point scale (Appendix). The 4 items ask how frequently during the previous 4 weeks the respondent experienced difficulty falling asleep, difficulty staying asleep, waking up several times per night, and waking up feeling tired and worn out after the usual amount of sleep. The response alternatives were: not at all (1), 1 to 3 days (2), 4 to 7 days (3), 8 to 14 days (4), 15 to 21 days (5), and 22 to 28 days (6). A dichotomous index was computed and coded as 1 if the respondents reported that any of the above sleep disturbances occurred 15 or more nights during the previous 4 weeks or as 0, if not. The selection of 15 nights as the cut-off point for sleep disturbance was based on criteria from the Diagnostic and Statistical Manual of Mental Disorder, Fourth Edition, Text Revision (DSM-IV-TR),^11^ which stipulate that difficulty maintaining/initiating sleep or nonrestorative sleep should be present for 3 or more nights per week for at least 1 month. A similar cut-off point for sleep disturbance was used in previous studies.^3^^,^^12^^,^^13^

The survey also incorporated the 12-item GHQ^5^ as a measure of psychiatric morbidity. This instrument contains 1 item that enquires about sleep loss due to worry over the preceding few weeks. There are 4 possible response alternatives: not at all (1), no more than usual (2), rather more than usual (3), and much more than usual (4). A score of 3 or 4 on this item is considered to indicate sleep disturbance,^14^ and the same threshold was used in this study.

### Statistical analysis

In the present analysis, we only included data on participants who responded to the questions on the sleep items and covariates (*n* = 7777; 79% women; mean age, 49 years). More specifically, we included all baseline participants, namely, those who had responded to at least 2 of the 4 JSQ sleep items and the GHQ-12 sleep item and had provided responses to the predictors of sleep disturbance. Those with missing data for more than 2 of the JSQ items were excluded from the analyses.

We first computed descriptive statistics and inter-item correlations between the items of the JSQ and the sleep question derived from the GHQ-12. A receiver operating characteristic (ROC) curve was constructed to illustrate the diagnostic accuracy of the single-item sleep measure against the reference test of the validated JSQ multiple-item measure.^15^^,^^16^ To determine whether the sleep measures had similar predictors, 2 logistic regression analyses were performed on the GHQ-12 item and the JSQ as dependent variables using the same estimation models adjusted for age. The final set of analyses used the JSQ as the reference test to identify respondents “misclassified” by the GHQ-12 item.

Separate logistic regression models were used to compare differences in characteristics (predictor variables) between respondents who were true positives (positive on both the JSQ and GHQ-12), true negatives (negative on both the JSQ and GHQ-12), false positives (negative on JSQ and positive on GHQ-12), and false negatives (positive on JSQ and negative on GHQ-12). More specifically, true positives were compared with false positives, and true negatives were compared with false negatives, in separate logistic regression models to examine determinants of misclassification.

Several known predictors of sleep disturbance, eg, sociodemographic variables,^17^^,^^18^ working conditions,^19^^,^^20^ and health-related variables,^21^^–^^23^ were used to identify the factors associated with sleep disturbance as measured by the GHQ-12 item and the JSQ. We also examined whether these factors could distinguish between participants with discrepant responses to the 2 sleep-disturbance measures. The sociodemographic variables were age (continuous from 40 to 60 years) and partnership status (unmarried vs married or cohabiting). The working-conditions variables that have been previously identified as important for sleep were physical working conditions (physical workload, based on factor analyses of 18 items, with factor loadings classified by quartiles of workload, from very low to very high),^24^ psychosocial working conditions (9 items on job control and 9 items on job demands, to assess low and high job demands and job control),^25^ and a single item on work–family interference (a 7-point scale, from very dissatisfied to very satisfied in combining paid work and family). The health-behaviors variables included current smoking (no/yes), heavy drinking (coded as no/yes, based on reported units of beer, wine, and spirits consumed during a typical week), and obesity (normal or overweight vs a body mass index ≥30 kg/m^2^, calculated from self-reported height and weight). We also examined self-rated mental and physical health (5-point scale ranging from excellent to very poor), history of angina pectoris based on the Rose questionnaire (no vs yes for those fulfilling all the original criteria).^26^ All categorical covariates were treated as continuous variables in the logistic regression models. Among the dichotomous variables, the reference category was “no”/“the advantaged situation”. The details of these items and their measurement have been previously reported.^8^^,^^19^^,^^27^^,^^28^ All analyses were stratified by sex. The analyses were done using SAS version 9.2 (SAS Institute, Cary, NC, USA).

## RESULTS

Only 7% of men and 8% of women were identified on both measures as having a sleep disturbance (true positives), whilst 11% of men and 12% of women were identified as false positives and 10% of men and 12% of women were identified as false negatives (Tables 1a and 1b). Specificity, ie, the proportion of true negatives (no sleep disturbance on either the reference [JSQ] or our test measure, the GHQ-12 sleep item), was 85.4% among women and 87.4% among men. Sensitivity, ie, the proportion of true positives (sleep disturbance on both measures), was 38.3% among women and 39.0% among men. Among women, positive predictive value was 39.4% (469/1192) and negative predictive value was 84.9% (4228/4983). Among men, the respective figures were 38.5% (105/273) and 87.7% (1165/1329).

**Table 1a.** Cross-tabulation of sleep measures among men (*n* = 1602)^a^

	**JSQ sleep disturbance**		
	Yes	No	
**Sleep loss due to worries (GHQ-12)**			
Yes	105	168	PPV 38.5%
No	164	1165	NPV 87.7%
	Sensitivity 39.0%	Specificity 87.4%	

PPV, positive predictive value; NPV, negative predictive value.

^a^The Helsinki Health Study.

**Table 1b.** Cross-tabulation of sleep measures among women (*n* = 6175)^a^

	**JSQ sleep disturbance**		
	Yes	No	
**Sleep loss due to worries (GHQ-12)**			
Yes	469	723	PPV 39.4%
No	755	4228	NPV 84.9%
	Sensitivity 38.3%	Specificity 85.4%	

PPV, positive predictive value; NPV, negative predictive value.

^a^The Helsinki Health Study.

Correlations between the items on the JSQ and the GHQ-12 sleep item varied from 0.39 to 0.47 among both women and men (Table 2). The highest correlation (0.47, *P* < 0.0001) was observed among men, between difficulties staying asleep and the GHQ-12 sleep item on sleep loss due to worry. The correlation between the summary score of all the JSQ items (range 4–24) and the GHQ-12 sleep item (range 1–4) was 0.52 (*P* < 0.0001).

**Table 2.** Spearman correlation coefficients between the JSQ items and GHQ-12 item^a^

	GHQ-12: sleep loss due to worries		
	
	All (*n* = 7777)	Men (*n* = 1602)	Women (*n* = 6175)
1) Difficulty falling asleep	0.44	0.43	0.44
2) Waking several times	0.39	0.40	0.39
3) Difficulty staying asleep	0.46	0.47	0.45
4) Nonrestorative sleep	0.40	0.42	0.40
5) Sleep disturbance 15–28 times per month vs others	0.26	0.26	0.26
6) Sleep disturbance approximately once a week or more frequently vs others	0.37	0.40	0.39
7) Sleep disturbance (JSQ score, 4–24)	0.52	0.53	0.52
*P-value (for all correlations) <0.0001*			

^a^Standardized Cronbach’s alpha for the 4 JSQ items and GHQ-12 item = 0.840; standardized Cronbach’s alpha for the 4 JSQ items = 0.843.

**Figure 1.**
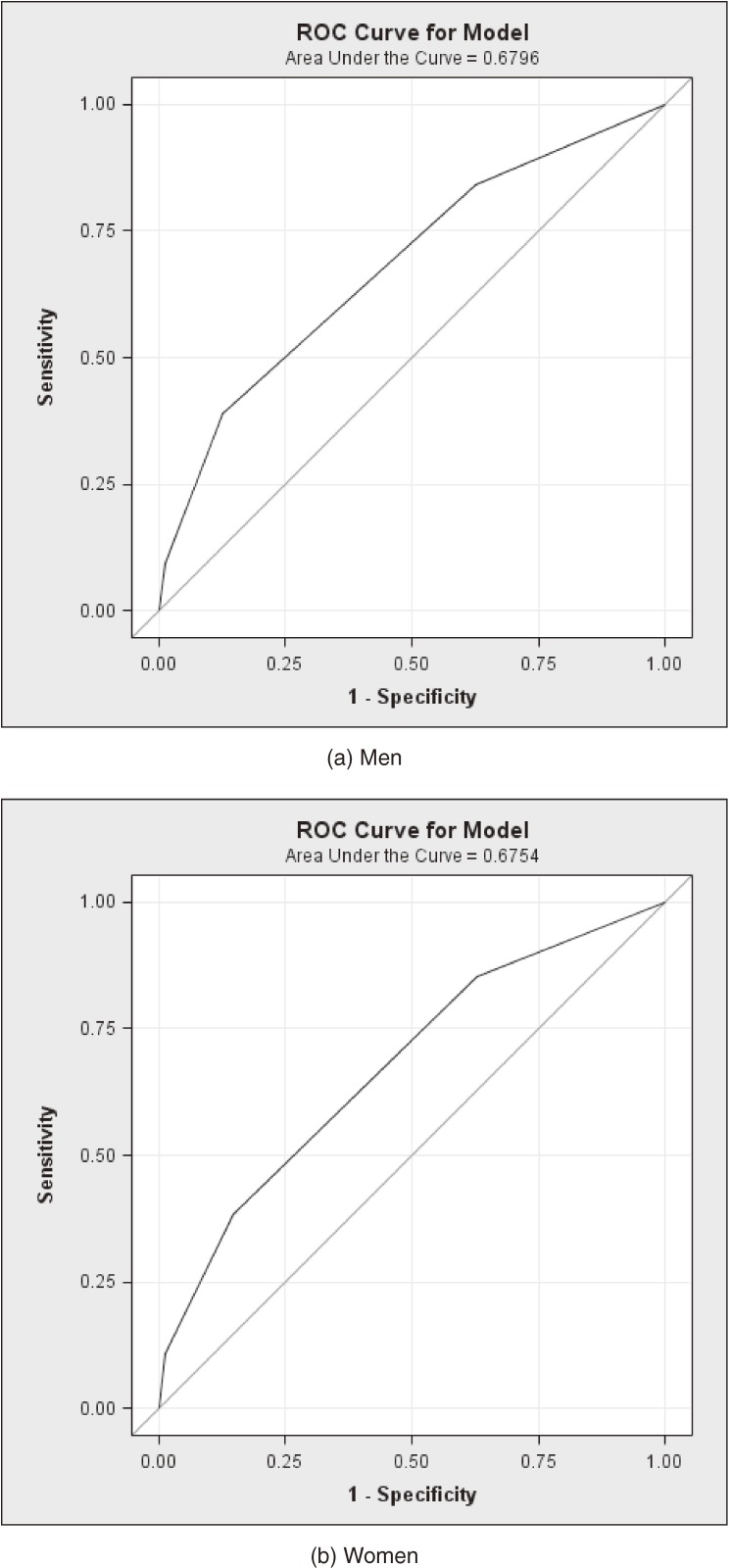
ROC curve comparing a dichotomized JSQ (any sleep problem 15–28 times during 4 weeks vs others) and the 4 categories of the GHQ-12 sleep item (range 1–4).

The ROC curve shows the extent to which the single GHQ-12 item accurately reproduced the JSQ (Figures 1a and 1b). The estimated area under the curve was 0.68 among both men and women for the curve comparing a dichotomized JSQ (any sleep problem occurring 15–28 times during a 4-week period vs others) and the 4 categories of the GHQ-12 sleep item (range 1–4). This value suggests that the ability of the GHQ-12 sleep item to discriminate true positives from false positives is modest.

Finally, in separate binary logistic regression analyses we investigated characteristics associated with sleep disturbance (Tables 3a and 3b). The GHQ-12 item and the JSQ showed mostly similar associations with sociodemographic factors, working conditions, and health-related factors among women and men.

**Table 3a.** Predictors of sleep disturbance^a^ by multi-item and single-item measures among men

	Sleep disturbance on GHQ-12 (*n* = 1602)		Sleep disturbance on JSQ (*n* = 1602)	
		
	OR	95% CI	OR	95% CI
Age (continuous, 40–60 years)	0.97	(0.95–0.99)	1.01	(0.99–1.03)

Marital status (ref = not living with a partner)	0.59	(0.44–0.79)	0.69	(0.51–0.93)

Education (ref = least education)	0.99	(0.84–1.15)	0.83	(0.71–0.97)

Heavy physical workload (ref = low-to-moderate physical workload)	1.92	(1.46–2.54)	1.85	(1.40–2.46)

Work stress				
High job demands (ref = low demands)	2.42	(1.85–3.16)	1.75	(1.34–2.27)
Low job control (ref = high control)	1.32	(1.01–1.71)	1.47	(1.13–1.92)

Work–family interference (ref = satisfied)	2.34	(1.90–2.87)	2.02	(1.64–2.48)

Health behavior and obesity				
Smoking (ref = nonsmoker)	1.36	(1.03–1.80)	1.12	(0.84–1.49)
Heavy drinking (ref = moderate drinker)	1.68	(1.08–2.62)	1.34	(0.84–2.13)
Obesity (ref = not obese)	1.26	(0.88–1.80)	1.66	(1.19–2.32)

Health				
Self-rated health (mental and physical health status; ref = excellent health)	2.15	(1.83–2.54)	2.51	(2.11–2.98)
Angina pectoris symptoms (ref = no history of angina)	2.23	(1.24–4.02)	1.66	(0.92–3.00)

^a^Adjusted for age.

**Table 3b.** Predictors of sleep disturbance^a^ by multi-item and single-item measures among women

	Sleep disturbance on GHQ-12 (*n* = 6175)		Sleep disturbance on JSQ (*n* = 6175)	
		
	OR	95% CI	OR	95% CI
Age (continuous, 40–60 years)	1.01	(1.00–1.02)	1.04	(1.03–1.05)

Marital status (ref = not living with a partner)	1.01	(0.88–1.15)	0.94	(0.82–1.07)

Education (ref = least education)	1.05	(0.97–1.14)	1.02	(0.95–1.11)

Heavy physical workload (ref = low-to-moderate physical workload)	1.40	(1.22–1.61)	1.71	(1.49–1.96)

Work stress				
High job demands (ref = low demands)	2.10	(1.84–2.39)	1.75	(1.54–1.99)
Low job control (ref = high control)	1.17	(1.03–1.33)	1.33	(1.18–1.51)

Work–family interference (ref = satisfied)	2.12	(1.92–2.33)	1.98	(1.80–2.19)

Health behavior and obesity				
Smoking (ref = nonsmoker)	1.32	(1.14–1.53)	1.16	(1.00–1.34)
Heavy drinking (ref = moderate drinker)	1.67	(1.33–2.10)	1.41	(1.12–1.78)
Obesity (ref = not obese)	1.05	(0.88–1.26)	1.19	(1.00–1.41)

Health				
Self-rated health (mental and physical health status; ref = excellent health)	2.11	(1.94–2.29)	2.56	(2.35–2.80)
Angina pectoris symptoms (ref = no history of angina)	2.19	(1.75–2.76)	1.77	(1.40–2.24)

^a^Adjusted for age.

We also examined predictors of misclassification by comparing separately the correctly classified respondents with the “false positives” and “false negatives” (data not shown). Respondents falsely identified by the GHQ-12 measure as having sleep disturbance (false positives) were less likely to report poor self-rated health, whereas respondents that the GHQ-12 missed (false negatives) were more likely to be women, to report dissatisfaction with work–family interference, and have poorer self-rated health. All other differences were small and nonsignificant.

## DISCUSSION

We examined whether a widely available single-item measure of sleep disturbance could be used as a suitable alternative to a multi-item sleep disturbance questionnaire (the JSQ). The clinical usefulness of a sleep scale depends on its ability to correctly identify the presence (sensitivity) or absence (specificity) of sleep disturbance. Our findings indicate that the sensitivity of the GHQ-12 item was relatively low (39%), although specificity was better (87%). These results suggest that the GHQ sleep item has a limited ability to correctly identify sleep disturbances, as compared with a multi-item sleep disturbance scale such as the JSQ. The relatively poor level of agreement between these measures was reflected in the ROC curve, which showed only modest discrimination (area under the curve, 0.68). However, there is often a trade-off between sensitivity and specificity.^29^ For instance, using the present data, the sensitivity of the GHQ item could be increased by choosing a different cut-off point (eg, 1 instead of 2). However, such an adjustment would lower the specificity of an item. In the present study, we were interested in determining the validity of the commonly used cut-off point for the GHQ sleep item. Future researchers might select a different cut-off point to match their varying goals, and the present findings could provide valuable guidance for such adjustment.

Self-rated health was the most consistent predictor of sleep disturbance on both measures although, among women, the associations appeared somewhat stronger for the JSQ. As the GHQ-12 sleep item specifically asked about sleep loss due to worry, it could have captured transient episodes that would not be identified in the context of mental or somatic illnesses. The JSQ, in turn, focused on frequent disturbance, which suggests a pathologic context.

This study used the JSQ as the reference test in evaluating the GHQ-12 item. This immediately limited the explanatory power of the study, as the JSQ might be unable to capture all sleep disturbances (as suggested by the considerable number of false positives revealed by the GHQ-12 item). Furthermore, the cut-off point used to identify ‘cases’ on the JSQ was any sleep disturbance that occurred on at least 15 days during the previous 4 weeks, which is a convention based on the current DSM-IV-TR criteria for insomnia.^11^ Thus, this cross-validation analysis showed that the single GHQ-12 item only modestly discriminates more severe symptoms, but may be better at capturing milder sleep disturbances. This is supported by our control analyses that used sleep disturbance occurring an average of once a week as a cut-off point (data not shown). The positive predictive values increased and negative predictive values were unchanged.

Previous studies indicated that, when comparing 2 different measures of the same construct, each might positively identify some people with sleep disturbances that would have been missed by the other measure.^30^ It is possible that the use of different cut-offs in the present study might have resulted in a closer association with the GHQ-12 item, but at the expense of over- or under-including the “reality” of sleep disturbance. Small correlations between the GHQ-12 and other measures of sleep disturbance, such as the PSQI, were found in prior studies,^31^ similar to the findings of the present study.

Can the single GHQ-12 item substitute for the JSQ as a measure of sleep disturbance? The answer must be no. That is not to say, however, that the GHQ-12 item has little or no value in sleep research. Rather, it might identify a partially differing aspect of the construct of sleep. The modest discrimination of the single GHQ-12 item, relative to the multi-item JSQ, is possibly unsurprising for a measure that assesses a particular facet of the multidimensional construct of sleep disturbance.

## APPENDIX

### JSQ for clinical research^2^ and an item on sleep from the GHQ-12^5^

How often during the previous 4 weeks did you have the following symptoms:

a) difficulty falling asleep?
b) waking up several times per night?
c) difficulty staying asleep (including waking up too early)?
d) waking up feeling tired and worn out after usual amount of sleep?

Response alternatives:

(1) not at all
(2) 1–3 nights
(3) 4–7 nights
(4) 8–14 nights
(5) 15–21 nights
(6) 22–28 nights

#### GHQ-12 sleep item

Have you recently lost much sleep due to worry (during the previous few weeks)?

1) not at all
2) no more than usual
3) rather more than usual
4) much more than usual
